# Emerging Paediatric Uses of Dupilumab Beyond Approvals

**DOI:** 10.1111/cea.70381

**Published:** 2026-06-23

**Authors:** Simone Foti Randazzese, Anna Calamita, Diletta D'Angelo, Alessia Marseglia, Esther Mesa Lombardero, Sara Pedersoli, Micol Tedeschi, Mara De Amici, Sara Manti, Valeria Brazzelli, Amelia Licari, Gian Luigi Marseglia

**Affiliations:** ^1^ Pediatric Unit, Department of Clinical, Surgical, Diagnostic and Pediatric Sciences University of Pavia Pavia Italy; ^2^ Pediatric Clinic Fondazione IRCCS Policlinico San Matteo Pavia Italy; ^3^ Hospital Universitario Central de Asturias (HUCA) Oviedo Asturias Spain; ^4^ Immuno‐Allergology Laboratory of Clinical Chemistry and Pediatric Clinic Fondazione IRCCS Policlinico San Matteo Pavia Italy; ^5^ Pediatric Unit, Department of Human Pathology in Adult and Developmental Age “Gaetano Barresi” University of Messina Messina Italy; ^6^ Dermatologic Clinic Fondazione IRCCS Policlinico San Matteo Pavia Italy; ^7^ Department of Clinical, Surgical, Diagnostic and Pediatric Sciences, Institute of Dermatology Università degli Studi di Pavia Pavia Italy

**Keywords:** dupilumab, off‐label use, paediatrics

## Abstract

Dupilumab is a fully human IgG4 monoclonal antibody that targets the interleukin‐4 receptor alpha subunit, thereby inhibiting interleukin‐4 and interleukin‐13 signalling, two central drivers of type‐2 (T2) inflammation. It is currently approved by both the Food and Drug Administration and the European Medicines Agency for multiple indications, including moderate‐to‐severe atopic dermatitis (from 6 months of age), add‐on maintenance therapy for moderate‐to‐severe asthma with T2 inflammation (≥ 6 years), eosinophilic esophagitis (≥ 1 year and ≥ 15 kg), chronic rhinosinusitis with nasal polyps (≥ 12 years in the United States and ≥ 18 years in Europe), and chronic spontaneous urticaria (≥ 12 years in the United States and ≥ 2 years in Europe) who remains symptomatic despite antihistamine therapy. Additional adult indications include prurigo nodularis, bullous pemphigoid, and chronic obstructive pulmonary disease with an eosinophilic phenotype. Beyond these established indications, increasing evidence suggests a broader therapeutic potential for dupilumab in paediatric diseases characterized by T2 immune dysregulation. This narrative review synthesizes the emerging literature on off‐label and investigational paediatric uses of dupilumab, focusing on inflammatory skin diseases, inborn errors of immunity with severe atopic phenotypes, transplant‐associated immune dysregulation, selected respiratory conditions and food allergy. Although current evidence largely derives from case reports, small case series, and observational studies, reported outcomes consistently indicate significant improvements in disease severity, and quality of life, with a favourable safety profile. These findings highlight the expanding role of IL‐4/IL‐13 blockade in paediatrics and underscore the need for prospective studies to better define efficacy, long‐term safety, and optimal patient selection in emerging indications.

## Key Points

- Dupilumab may be effective in selected paediatric diseases characterized by T2 inflammation beyond approved indications.
- Treatment response appears heterogeneous, with improved outcomes in patients exhibiting atopic or T2 immune signatures.
- Evidence remains limited to small, heterogeneous studies; well‐designed prospective trials are warranted.

## Graphical abstract

Dupilumab, through IL‐4Rα blockade, shows promising efficacy beyond approved indications in paediatric diseases driven by T2 inflammation. Emerging evidence—mainly from small studies—supports improvements in disease severity and quality of life, highlighting its potential as a targeted, steroid‐sparing therapy while underscoring the need for prospective trials.

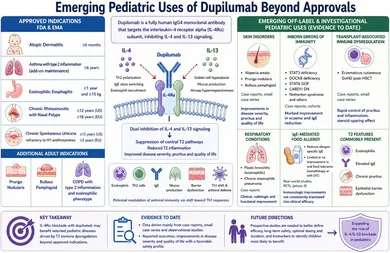

## 1 Introduction

Dupilumab is a fully human IgG4 monoclonal antibody targeting the interleukin (IL)‐4 receptor alpha (IL‐4Rα) subunit, a shared component of the receptor complexes for IL‐4 and IL‐13, two key cytokines driving type 2 (T2) inflammation [1, 2]. By binding IL‐4Rα, dupilumab inhibits signalling through both the type I receptor (IL‐4Rα/γc), predominantly active in haematopoietic cells, and the type II receptor (IL‐4Rα/IL‐13Rα1), which mediates IL‐4 and IL‐13 signalling in structural and epithelial tissues [3]. This dual inhibition suppresses central T2 immune pathways, including T‐helper (Th)2 differentiation, immunoglobulin E (IgE) class switching, eosinophil recruitment, and production of pro‐inflammatory cytokines and chemokines [4, 5]. Beyond its anti‐inflammatory effects, modulation of the IL‐4/IL‐13 axis may influence antiviral immunity. By attenuating Th2‐skewed responses, dupilumab may theoretically favour a relative shift toward Th1 immune responses, with potential implications for interferon‐γ production and antiviral defence; however, the clinical relevance of this effect remains incompletely understood, particularly in paediatric populations [6, 7, 8].

IL‐4 and IL‐13 have complementary and partially overlapping roles in T2 inflammatory diseases. IL‐4 is critical for Th2 polarization, B‐cell activation, IgE class switching, and eosinophil trafficking, whereas IL‐13 primarily affects epithelial and structural cells, promoting goblet‐cell hyperplasia, mucus production, airway hyperresponsiveness, and tissue remodelling [9, 10, 11]. Both cytokines induce epithelial and endothelial release of chemokines such as eotaxin‐3, amplifying eosinophilic inflammation [12]. Due to this functional redundancy, targeting a single cytokine often results in incomplete disease control, whereas IL‐4Rα blockade simultaneously disrupts multiple downstream pathways [13]. Accordingly, the IL‐4/IL‐13 axis is considered a central driver of diseases characterized by epithelial barrier dysfunction, eosinophilia, chronic pruritus, and systemic immune dysregulation [14, 15].

Since its approval in 2017, dupilumab has gained multiple indications [16]. It is approved by the Food and Drug Administration (FDA) and European Medicines Agency (EMA) for moderate‐to‐severe atopic dermatitis (AD) (≥ 6 months), asthma (≥ 6 years) with T2 inflammation or oral corticosteroid dependence, and eosinophilic esophagitis (≥ 1 year, ≥ 15 kg). It is also indicated for chronic rhinosinusitis with nasal polyps as add‐on therapy, with broader age approval by the FDA (≥ 12 years) than the EMA (primarily adults). Dupilumab is approved for chronic spontaneous urticaria in patients refractory to H1‐antihistamines (FDA ≥ 12 years; EMA ≥ 2 years) (Figure 1). Adult‐only indications include prurigo nodularis, bullous pemphigoid (FDA), and chronic obstructive pulmonary disease with T2 inflammation and an eosinophilic phenotype [17, 18].

**FIGURE 1:**
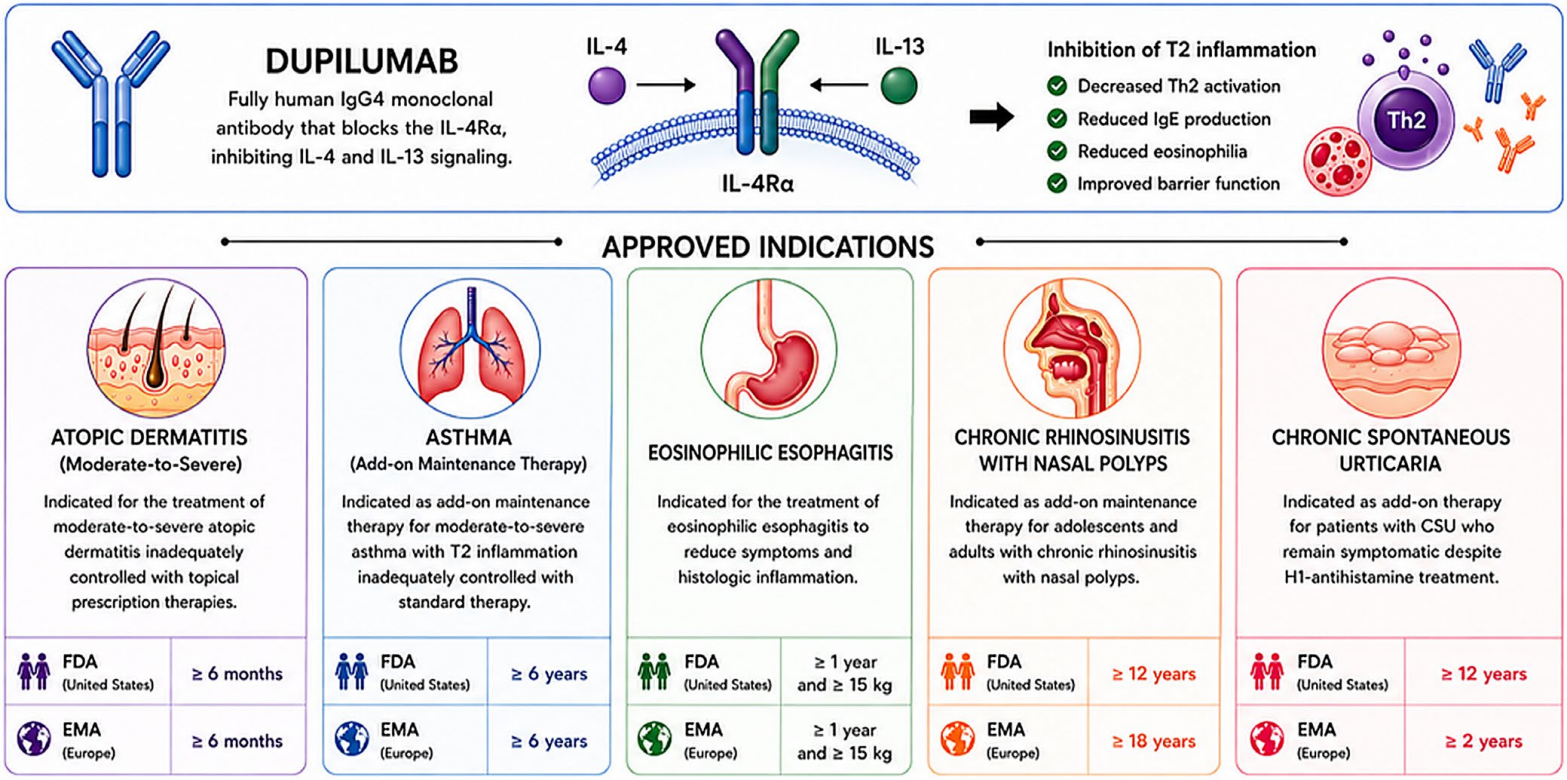
Approved indications of dupilumab in children and adolescents according to FDA and EMA. Current regulatory approvals of dupilumab in paediatric patients, highlighting age eligibility and differences between FDA and EMA indications across T2‐mediated diseases (CSU, chronic spontaneous urticaria; EMA, European Medicines Agency; FDA, Food and Drug Administration; IgE, immunoglobulin E; IgG, immunoglobulin G; IL, interleukin; IL‐4Rα, interleukin‐4 receptor alpha subunit; T2, type 2; Th2, T‐helper 2).

Across approved paediatric indications, dupilumab has shown a favourable benefit–risk profile, with safety findings remaining largely consistent across randomized clinical trials (RCTs), long‐term extension studies, and real‐world cohorts. The most frequently reported adverse events are injection‐site reactions, conjunctivitis, transient eosinophilia, and herpesvirus infections. Serious treatment‐related adverse events are uncommon, and discontinuation rates due to adverse events remain low. Rare cases of eosinophilic complications and paradoxical inflammatory conditions have been reported, warranting clinical vigilance during treatment [2, 13].

Emerging evidence suggests that T2 immune dysregulation may contribute to paediatric diseases not traditionally classified as T2‐mediated (Figure 2). Consequently, dupilumab is increasingly explored in off‐label settings, including dermatologic conditions such as alopecia areata (AA), PN, and BP, inborn errors of immunity (IEI) with atopic phenotypes, transplant‐associated conditions such as AD–like graft‐versus‐host disease (GvHD), and rare eosinophilic or allergic disorders involving the respiratory and gastrointestinal systems. Although dupilumab offers a targeted approach with a favourable safety profile compared to systemic immunosuppressants, current evidence remains limited, largely derived from case reports, small case series, and retrospective studies.

**FIGURE 2:**
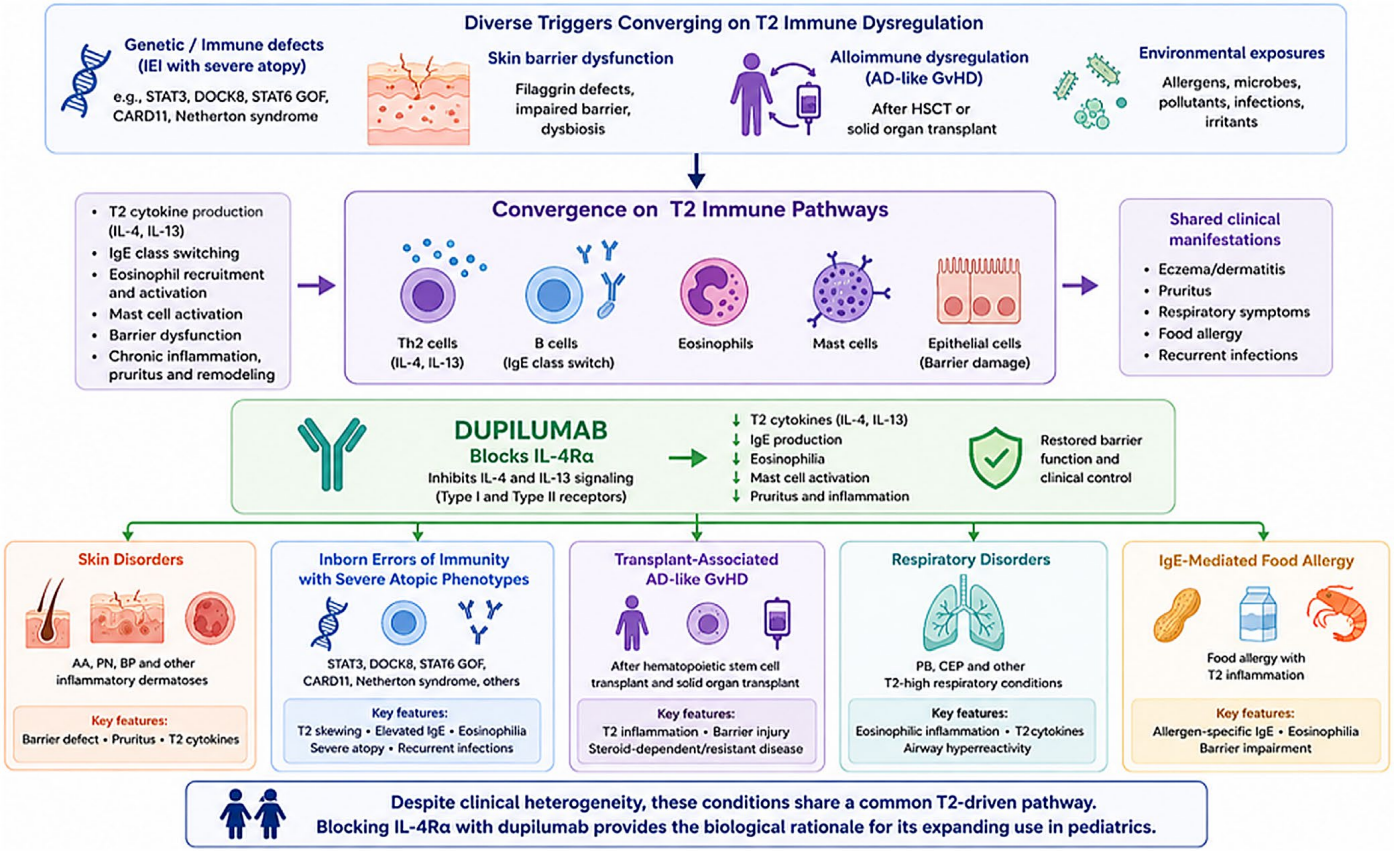
Type 2 immune dysregulation as the common therapeutic target of emerging paediatric dupilumab indications. Overview of the shared T2 inflammatory pathways linking diverse emerging paediatric indications and providing the biological rationale for IL‐4Rα blockade beyond currently approved diseases (AA, alopecia areata; AD, atopic dermatitis; BP, bullous pemphigoid; CEP, chronic eosinophilic pneumonia; GOF, gain‐of‐function; GvHD, graft‐versus‐host disease; HSCT, haematopoietic stem cell transplantation; IEI, inborn errors of immunity; IgE, immunoglobulin E; IL, interleukin; PB, plastic bronchitis; PN, prurigo nodularis; T2, type 2).

This review synthesizes available evidence on the off‐label and investigational use of dupilumab in paediatric populations beyond approved indications. For the purposes of this review, paediatric patients were defined as individuals younger than 18 years of age. Studies including mixed paediatric and adult populations were considered when paediatric data were reported separately or when the study population predominantly comprised children and adolescents. Relevant studies were identified through a PubMed search of English‐language publications from January 1, 2005, to December 31, 2025, using Medical Subject Headings and free‐text terms including ‘dupilumab’, ‘IL‐4Rα blockade’, ‘T2 inflammation’, ‘paediatric’, ‘children’, ‘adolescent’, ‘alopecia areata’, ‘prurigo nodularis’, ‘bullous pemphigoid’, ‘inborn errors of immunity’, ‘primary immunodeficiency’, ‘graft‐versus‐host disease’, ‘respiratory eosinophilic disorders’, ‘plastic bronchitis’, ‘chronic eosinophilic pneumonia’, and ‘food allergy’. RCTs, observational studies, registries, retrospective cohorts, case series, and case reports were eligible for inclusion. Publications focused exclusively on approved paediatric indications of dupilumab, non‐paediatric populations without relevant paediatric data, non‐English articles, conference abstracts without sufficient clinical information, and duplicate reports were excluded. Additional studies were identified through manual screening of the reference lists of selected articles. The available evidence was subsequently organized and synthesized thematically according to clinical disease categories. No formal meta‐analysis was performed because of substantial heterogeneity in study design, patient populations, and reported outcome measures.

## 2 Skin Disorders

Increasing evidence indicates that T2 inflammatory pathways contribute to the pathogenesis of several dermatologic conditions not traditionally classified as Th2‐mediated [19, 20]. This has stimulated interest in IL‐4/IL‐13 blockade as a therapeutic strategy for paediatric skin diseases characterized by immune dysregulation, chronic pruritus, or epithelial barrier dysfunction. Among these, AA, PN, and BP have emerged as the most frequently reported conditions in which dupilumab has been explored.

### 2.1 Alopecia Areata

AA is a non‐scarring autoimmune hair loss disorder affecting approximately 2% of the general population, including children [21]. Although classically considered a Th1/Th17‐mediated disease, increasing evidence suggests that T2 immune activation may contribute to disease expression in a subset of patients, particularly those with concomitant atopic disorders [22, 23]. Elevated serum IgE levels, peripheral eosinophilia, and enhanced IL‐4/IL‐13 signalling have been reported in both adult and paediatric patients with AA, especially when associated with AD, providing a biological rationale for IL‐4Rα blockade [24, 25, 26, 27].

Evidence supporting dupilumab in paediatric AA remains limited and heterogeneous, consisting mainly of case reports, small case series, and retrospective studies [28, 29]. Both clinical improvement and paradoxical onset or worsening of AA during dupilumab therapy have been reported, highlighting the immunologic heterogeneity of the disease and the likelihood of endotype‐specific responses [28, 29].

The largest synthesis of paediatric data were provided by Metko et al., who reported clinically significant hair regrowth in 77.4% of 31 children (mean age 11.4 years) with AA and concomitant AD, with a mean reduction in Severity of Alopecia Tool (SALT) scores of 42.6% (*p* < 0.01) after a mean treatment duration of 3.2 months [29]. Similarly, in the largest retrospective paediatric series, David et al. evaluated 20 children aged 5–16 years with refractory AA and moderate‐to‐severe AD and observed a significant reduction in mean SALT scores from 54.4 at baseline to 20.4 at Week 48 and 2.2 beyond 72 weeks (*p* < 0.01). Higher baseline IgE levels and lower initial SALT scores were associated with greater treatment responses, supporting the hypothesis that T2‐dominant disease may predict improved outcomes [30].

Additional retrospective studies have reported clinically meaningful hair regrowth in paediatric patients, particularly in those with concomitant AD [31, 32], whereas responses appear less favourable in longstanding alopecia universalis or ophiasis patterns [33].

Individual case reports have also described complete or near‐complete hair regrowth in severe or treatment‐resistant paediatric AA, including patients previously unresponsive to Janus kinase (JAK) inhibitors [34, 35, 36]. Additional reports documented successful treatment of ophiasis‐pattern AA, partial responses requiring subsequent baricitinib therapy, and hair repigmentation accompanying regrowth [37, 38, 39]. Although these observations are clinically interesting, they remain anecdotal and should be regarded as hypothesis‐generating rather than conclusive evidence.

Overall, these findings should be interpreted cautiously. Most reported patients received dupilumab primarily for AD rather than AA, introducing potential selection bias and limiting generalizability to non‐atopic AA populations. Furthermore, reports of new‐onset or worsening AA during treatment suggest that only selected immunological endotypes may benefit [40, 41, 42]. Overall, current evidence indicates that dupilumab may represent a promising option for paediatric AA, particularly in patients with concomitant AD or other markers of T2 inflammation. Ongoing clinical trials, including NCT05866562, are expected to better define its efficacy, predictors of response, and role in the paediatric AA treatment landscape [43].

### 2.2 Prurigo Nodularis

PN is a chronic inflammatory dermatosis characterized by intensely pruritic, hyperkeratotic nodules sustained by a self‐perpetuating itch–scratch cycle [44].

Although more common in adults, PN is also recognized in paediatric populations. A U.S. claims‐based study estimated a paediatric prevalence of 21.6 per 100,000, indicating that while uncommon, the disease is clinically relevant [45]. Paediatric PN is frequently associated with atopic conditions and can significantly impair sleep, psychological well‐being, and quality of life (QoL) [46].

The pathophysiology of PN involves complex interactions between immune dysregulation, neuronal sensitization, and barrier dysfunction. T2 cytokines play a central role by directly sensitizing peripheral sensory neurons, amplifying neurogenic inflammation, promoting IgE production, and enhancing recruitment of inflammatory cells to the skin [47, 48]. However, PN is increasingly recognized as a neuroimmune disorder involving multiple interconnected pathways. IL‐31, a key pruritogenic cytokine produced by Th2 cells, directly activates sensory neurons and contributes to chronic itch. Neurogenic mediators such as substance P further amplify inflammation and pruritus through bidirectional communication between immune cells and peripheral nerves. In addition, activation of the JAK–STAT signalling pathway mediates downstream effects of several cytokines implicated in PN, including IL‐4, IL‐13, and IL‐31, thereby contributing to persistent inflammation and maintenance of the itch–scratch cycle. These mechanisms collectively provide a strong rationale for targeting the IL‐4/IL‐13 axis, particularly in atopic individuals, while also highlighting the multifactorial nature of PN pathogenesis [49].

Standard management in children focuses on controlling pruritus and interrupting the itch–scratch cycle. First‐line therapy typically includes potent topical corticosteroids, calcineurin inhibitors, emollients, and behavioural interventions. Oral antihistamines are commonly used, particularly for nocturnal symptoms, although efficacy is often limited. Phototherapy and systemic agents such as cyclosporine may be required in refractory cases, but long‐term tolerability remains a concern [50].

Robust evidence for dupilumab in PN derives from adult populations. In the phase III PRIME and PRIME2 trials, dupilumab significantly improved pruritus and skin lesions compared with placebo in moderate‐to‐severe PN, leading to regulatory approval for adult patients [51].

Paediatric data, however, remain limited to case reports. Fachler et al. described marked improvement in a 9‐year‐old girl [52], and Giovannini et al. reported successful treatment in an adolescent patient [53]. The most detailed paediatric case, reported by Zhang et al. involved an 11‐year‐old with severe atopic PN and allergic rhinitis refractory to multiple therapies, including JAK1 inhibition. Dupilumab led to rapid pruritus reduction within 2 weeks and substantial clinical and QoL improvement by Week 4, with sustained remission after treatment discontinuation [54]. Additional reports, including a 5‐year‐old child, suggest that benefit may extend to younger age groups [55].

Across available reports, dupilumab was well tolerated, with no serious adverse events described. Although evidence remains anecdotal, the consistency of rapid antipruritic effects and progressive lesion improvement is notable. An ongoing open‐label phase III paediatric study (NCT06293053) will provide further data on pharmacokinetics and safety in patients aged 6 months to < 18 years [56]. At present, dupilumab appears to be a promising option for selected children with refractory PN, particularly in the presence of atopy or severe pruritus.

### 2.3 Bullous Pemphigoid

BP is the most common autoimmune subepidermal blistering disease, typically affecting older adults [57, 58]. Paediatric BP is rare, accounting for approximately 1% of cases, with fewer than 100 cases reported worldwide [59]. Despite its rarity, it may be associated with significant morbidity and is likely underdiagnosed due to atypical or non‐bullous presentations mimicking urticaria, eczema, or prurigo [60, 61].

BP is mediated by autoantibodies directed against hemidesmosomal proteins, primarily BP180 (type XVII collagen) and BP230. Binding of these autoantibodies to the basement membrane zone triggers complement activation and recruitment of inflammatory cells, particularly eosinophils, leading to dermoepidermal separation and blister formation. Increasing evidence implicates T2 cytokines, including IL‐4, IL‐5, and IL‐13, in disease pathogenesis, contributing to eosinophilic inflammation and pruritus [62], thereby providing a mechanistic rationale for IL‐4Rα blockade.

Standard treatment includes high‐potency topical corticosteroids, systemic corticosteroids for more extensive disease, and steroid‐sparing agents such as methotrexate, azathioprine, or mycophenolate mofetil. Additional options include doxycycline, dapsone, intravenous immunoglobulins, and biologic agents in refractory cases [63]. In adults, retrospective studies and clinical trials, including ADEPT, suggest that dupilumab improves disease control, reduces pruritus, and exerts a corticosteroid‐sparing effect [64].

Paediatric evidence remains limited to isolated case reports. Almassio et al. described a 3‐year‐old girl with BP associated with multiple comorbidities, including cow's milk allergy, inflammatory bowel disease, thyroiditis, and immunodeficiency. Following an incomplete response to systemic corticosteroids and intravenous immunoglobulin, dupilumab induced complete remission of pruritus and blistering within 4 months. The patient remained in sustained remission at 18 months, with only mild conjunctivitis reported [59]. Although anecdotal, this observation supports further investigation of dupilumab in paediatric BP, particularly in complex cases with eosinophilic or atopic immune features.

## 3 Primary Immunodeficiencies and Immune Dysregulation Disorders

Several IEI present with prominent atopic features, including severe eczema, pruritus, hyper‐IgE, eosinophilia, and recurrent infections [65, 66]. Despite genetic heterogeneity, many converge on dysregulated T2 immunity, either due to impaired immune regulation, as in STAT3 or DOCK8 deficiency, or constitutive activation of T2 pathways, as in STAT6 gain‐of‐function (GOF) disease [67, 68]. These alterations frequently produce severe AD–like phenotypes refractory to conventional therapies [69]. Evidence supporting dupilumab in IEI is limited but growing, largely consisting of case reports, small case series, and one nationwide cohort study.

Autosomal dominant STAT3 hyper‐IgE syndrome exemplifies T2 dysregulation in IEI. Loss‐of‐function mutations impair IL‐6, IL‐10, and IL‐21 signalling, leading to defective Th17 differentiation, recurrent infections, and severe eczema with markedly elevated IgE [70]. In a reported 9‐year‐old patient, dupilumab induced complete remission of eczema within 20 weeks, with marked reductions in disease severity scores and substantial IgE decline [71].

DOCK8 deficiency, an autosomal recessive combined immunodeficiency, is characterized by severe atopy, recurrent infections, autoimmunity, and malignancy risk [72]. Impaired cytoskeletal organization and immune synapse formation lead to defective T‐cell survival, dysfunctional regulatory T cells, and Th2 predominance, resulting in extreme IgE elevation and severe AD [73]. Haematopoietic stem cell transplantation (HSCT) is the only curative option, but disease control prior to transplantation is critical [74]. Dupilumab has demonstrated rapid and sustained improvement in several paediatric cases, including resolution of eczema herpeticum and marked reductions in IgE levels, and has been successfully used as a bridge to HSCT [75, 76, 77].

STAT6 GOF variants represent a distinct mechanism of T2 hyperactivation. Constitutive STAT6 signalling enhances IL‐4 production and IL‐4Rα expression, driving profound Th2 polarization with severe eczema, FA, eosinophilia, and gastrointestinal involvement [78]. In a reported adolescent with refractory disease, dupilumab induced rapid and sustained remission of both cutaneous and gastrointestinal symptoms, despite prior anti–IL‐5 therapy. Long‐term follow‐up demonstrated durable disease control, marked reductions in IgE, and no increased infection risk [79].

Similarly, CARD11‐associated dominant‐negative disease leads to impaired NF‐κB signalling and a pronounced Th2‐skewed phenotype, including severe AD, asthma, and FA [80]. In a multicenter series including children aged 9–12 years with severe refractory AD, dupilumab induced rapid improvement within 4–12 weeks, with IGA decreasing from 3–4 to 0–1 and Eczema Area and Severity Index (EASI) reductions > 80%. In another case, SCORing Atopic Dermatitis (SCORAD) decreased from 84 to 40 within 4 months, enabling discontinuation of systemic therapy. Immunologic parameters improved, with eosinophils declining from ~1200–1470 to 500–800 cells/μL and IgE from > 2000–4080 to 296–687 IU/mL. Sustained control was observed up to 4 years, with good tolerability and no significant safety concerns [81].

Similar benefit has been described in trichothiodystrophy‐associated inflammatory skin disease, where dupilumab improved skin inflammation, pruritus, hair, nails, and QoL [82, 83, 84].

The most informative dataset comes from the French multicenter cohort by Guillemin et al., which included 37 patients with IEI‐associated eczema (median age 16 years). Most had combined immunodeficiencies, with STAT3 and DOCK8 defects particularly represented. Dupilumab showed a favourable safety profile: adverse events occurred in 35.1%, mainly mild ocular complications; infections were usually mild; discontinuation due to adverse effects was rare. Efficacy was encouraging, with 89.2% showing improvement at 3 months and 62.5% of those continuing therapy achieving complete remission by 12 months. Response was better in STAT3 deficiency than in DOCK8 deficiency (*p* = 0.032), suggesting biologic differences in treatment susceptibility [85].

Netherton syndrome (NS), a rare autosomal recessive genodermatosis with an estimated incidence of approximately 1 in 100,000–200,000 live births, further illustrates the role of T2 inflammation in non‐classical IEI [86, 87]. Caused by SPINK5 mutations encoding LEKTI, NS is characterized by severe skin barrier dysfunction due to uncontrolled epidermal protease activity. This activates the KLK5–PAR2–TSLP axis, promoting Th2 polarization and chronic inflammation [88, 89, 90, 91, 92]. Clinically, NS presents with erythroderma, ichthyosis, hair shaft abnormalities such as trichorrhexis invaginata (‘bamboo hair’), elevated IgE, severe pruritus, recurrent infections, and growth impairment [88, 89, 90].

Evidence for dupilumab in paediatric NS derives from small case series and reports. Treatment regimens were heterogeneous, including both weight‐based dosing (approximately 6–16 mg/kg) and fixed‐dose approaches (160–600 mg loading doses followed by maintenance every 2–4 weeks), with treatment durations extending from several months to over 2 years. In selected cases, dupilumab was administered in combination with adjunctive therapies such as intravenous immunoglobulin, particularly in patients with severe systemic involvement or immunologic dysregulation [86, 87, 88, 89, 90, 93, 94].

Several reports describe dramatic reductions in pruritus within days to weeks of treatment initiation, often accompanied by normalization of sleep patterns, reduced reliance on antihistamines or topical corticosteroids, and marked improvements in QoL [88, 89, 90].

Cutaneous responses, while generally favourable, appear more variable and phenotype‐dependent. Significant improvements in erythema and eczematous inflammation have been documented, with reductions in disease severity scores (e.g., EASI, SCORAD) typically ranging between 40% and 80% [87, 88, 89, 90]. However, structural features of NS demonstrate less predictable responses, with partial improvement in some patients and minimal change in others. This variability likely reflects the persistence of the underlying keratinization defect despite effective suppression of Th2‐driven inflammation.

Importantly, several paediatric case reports highlight multidimensional clinical benefits beyond skin inflammation. These include improvement in hair shaft abnormalities such as trichorrhexis invaginata, increased hair density, and partial normalization of hair morphology [95]. Additionally, reductions in the frequency and severity of cutaneous infections have been described, alongside improvements in growth parameters and nutritional status, suggesting a broader impact on systemic disease burden [86, 89]. In very young patients, including infants as young as 9 weeks of age, dupilumab has been associated with rapid and profound disease control without safety concerns, underscoring its potential utility even in early life [93].

From an immunological perspective, most reports document substantial reductions in serum IgE levels and, in some cases, eosinophil counts, although these changes are not uniformly correlated with clinical response [87, 92]. Emerging data also suggest that dupilumab may contribute to normalization of epidermal barrier–related pathways and modulation of the skin microbiome, further supporting its role in partially restoring cutaneous homeostasis despite the absence of direct correction of LEKTI deficiency [91, 96].

## 4 Graft‐Versus‐Host Disease and Transplant‐Associated T2 Inflammation

The use of dupilumab in paediatric GvHD has been described primarily in a distinctive cutaneous phenotype often referred to as AD–like or eczematous GvHD. Although the available evidence is limited to case reports and small case series, the reported clinical responses are notable, suggesting that T2‐targeted therapy may be beneficial in carefully selected post‐transplant patients.

GvHD is a major complication of allogeneic HSCT, with skin representing one of the most frequently involved organs [97]. Acute GvHD typically presents with erythematous maculopapular eruptions arising within the first 100 days post‐transplant, whereas chronic GvHD encompasses a broader spectrum of manifestations, including lichenoid, sclerodermoid, poikilodermatous, and eczematous phenotypes that may occur later in the disease course [98, 99]. In this setting, cutaneous inflammation is multifactorial, reflecting donor alloreactivity, tissue injury, impaired barrier function, immune reconstitution, infections, and exposure to immunosuppressive therapies [100].

The AD‐like phenotype, although increasingly recognized, remains poorly standardized. Clinically, it is characterized by diffuse erythema, xerosis, scaling, severe pruritus, eosinophilia, and elevated IgE levels. Histopathological features often include spongiosis and acanthosis, resembling AD, with occasional apoptotic keratinocytes suggestive of overlapping GvHD pathology. These patients frequently require prolonged systemic immunosuppression and are prone to relapse during treatment tapering [101].

Standard management of GvHD relies on systemic corticosteroids, with second‐line options including ruxolitinib, sirolimus, mycophenolate mofetil, rituximab, and extracorporeal photopheresis [101, 102]. However, these therapies are often only partially effective and are associated with an increased risk of infection. In contrast, dupilumab offers a more phenotype‐directed approach in patients with prominent T2 features. This is mechanistically plausible, as eczematous GvHD may be enriched for eosinophilia, elevated IgE, and Th2/Th17/Th22 immune signatures overlapping with AD [102, 103].

In a case series of four children aged 4–17 years with skin‐limited, treatment‐refractory GvHD following HSCT, dupilumab led to clinically meaningful improvement in three patients (75%), with reductions in inflammation and pruritus and improvement in IGA scores from 3–4 to 0 in responders. Follow‐up ranged from 18 to 26 months, during which dupilumab enabled discontinuation of systemic immunosuppression in all responders. One patient subsequently required ruxolitinib for extracutaneous GvHD, although cutaneous disease remained controlled. No significant infectious or other adverse events were reported [101].

Subsequent reports have corroborated these findings. A 26‐month‐old boy with Wiskott–Aldrich syndrome developed severe refractory eczematous dermatitis after HSCT during cyclosporine tapering; dupilumab induced rapid near‐complete remission and allowed withdrawal of cyclosporine without relapse [104]. In another series of four children aged 2–8 years with severe AD‐like GvHD refractory to multiple systemic therapies, dupilumab resulted in rapid improvement in pruritus and near‐complete or complete clearance within weeks to months, with marked reductions in Eczema Area and Severity Index scores (e.g., from 31.5 to 1.2, 38 to 0, and 17 to 2.6), as well as parallel improvements in body surface area involvement and Children's Dermatology Life Quality Index scores (e.g., from 26 to 1 and from 25 to 0) [105]. Additional reports have described similar steroid‐sparing efficacy in children with complex transplant histories, including mucopolysaccharidosis type I and multisystem chronic GvHD [106, 107]. A further small series of three HSCT recipients (school‐aged to adolescent) likewise documented rapid clinical improvement and sustained remission, allowing discontinuation of concomitant therapies without relapse [108].

Across reports, several consistent features emerge: severe steroid‐dependent or refractory disease, intense pruritus, substantial QoL impairment, frequent eosinophilia and elevated IgE levels, and histopathological evidence of spongiotic dermatitis. Notably, dupilumab was typically introduced late in the disease course, after multiple treatment failures, yet often resulted in rapid symptom control and facilitated tapering or discontinuation of broader immunosuppressive regimens.

Although the level of evidence remains low, the consistency of these observations suggests that dupilumab may represent a valuable steroid‐sparing option in paediatric AD‐like GvHD when clinical, laboratory, and histological features indicate predominant T2 immune activation. Prospective studies are needed to better define diagnostic criteria, optimal timing of initiation, treatment duration, and long‐term safety in this vulnerable patient population.

## 5 Respiratory Disorders

Experience with dupilumab in rare paediatric respiratory diseases remains extremely limited; however, available reports suggest potential benefit in selected conditions characterized by eosinophilic or T2–driven pulmonary inflammation. The most illustrative examples to date are plastic bronchitis (PB) and chronic eosinophilic pneumonia (CEP).

PB is characterized by the formation of obstructive bronchial casts that can lead to severe respiratory compromise. It occurs predominantly in children and has been associated with congenital heart disease, lymphatic abnormalities, asthma, infections, malignancy, and inflammatory airway disorders [109]. Histologically, bronchial casts are classified as inflammatory (type I), containing fibrin and inflammatory cells including eosinophils, or acellular (type II), more commonly observed in congenital heart disease [110, 111]. The eosinophilic inflammatory subtype is of relevance to dupilumab.

Briski et al. described a 14‐year‐old girl with severe uncontrolled asthma and recurrent eosinophilic PB complicated by pneumonia. Histological analysis of the casts revealed abundant eosinophils and Charcot–Leyden crystals, consistent with marked eosinophilic T2 inflammation. The patient was refractory to multiple therapeutic interventions, including bronchoscopic cast removal, systemic corticosteroids, inhaled therapies, bronchodilators, mucolytics, and bronchoscopic cryotherapy. Following initiation of dupilumab, she achieved sustained remission, with no further episodes of cast formation or pneumonia over 1 year of follow‐up, accompanied by significant improvement in lung function. Although limited to a single case, this report provides a compelling proof of concept [112].

CEP is a rare eosinophilic lung disease characterized by respiratory symptoms, pulmonary infiltrates, and peripheral eosinophilia, frequently in association with asthma. Although typically responsive to systemic corticosteroids, relapses during dose tapering are common, often resulting in prolonged steroid exposure [113, 114, 115].

Paediatric CEP is uncommon but well recognized. In a reported case of an 11‐year‐old girl with relapsing, steroid‐dependent CEP and progressive structural lung damage, including bronchiectasis, dupilumab was initiated in combination with cyclosporine. This resulted in rapid clinical, radiologic, and functional improvement. Cyclosporine was subsequently discontinued, and remission was maintained with dupilumab monotherapy without reported adverse effects [116].

Overall, the current evidence in paediatric respiratory disease remains anecdotal. Nevertheless, the biological rationale for IL‐4Rα blockade is strong in eosinophilic, asthma‐associated, steroid‐dependent pulmonary conditions, and further investigation in well‐designed prospective studies is warranted.

## 6 IgE‐Mediated Food Allergy

FA represents an increasingly significant paediatric health concern [117]. IgE‐mediated FA is characterized by reproducible hypersensitivity reactions to specific food proteins, ranging from urticaria and angioedema to respiratory compromise and anaphylaxis [118]. In the United States, approximately 5.3% of children are affected by diagnosed FA or related allergic conditions [119].

The pathophysiology of IgE‐mediated FA is closely linked to T2 immunity [120, 121]. During the sensitization phase, allergen‐specific Th2 cells secrete IL‐4 and IL‐13, promoting B‐cell activation, IgE class switching, and sustained production of allergen‐specific IgE. These antibodies bind to the high‐affinity FcεRI receptor on mast cells and basophils, and subsequent allergen re‐exposure induces cellular degranulation and acute allergic responses [122, 123]. By targeting IL‐4Rα, dupilumab may theoretically reduce allergen‐specific IgE production, attenuate the T2 inflammatory milieu, and facilitate the development of immune tolerance or desensitization [124, 125, 126].

However, current evidence remains heterogeneous and overall modest. Retrospective real‐world studies in paediatric patients treated with dupilumab for AD and concomitant FA consistently demonstrate progressive reductions in food‐specific IgE levels.

In the cohort reported by Emerson et al., which included 60 children aged 6 months to 18 years, food‐specific IgE levels declined significantly over time (*p* < 0.001), with an overall reduction exceeding 60%. Despite this, skin prick test reactivity remained unchanged (*p* = 0.8), and outcomes of oral food challenges were comparable to those observed in biologic‐naïve real‐world cohorts, indicating that immunologic improvements did not translate into clear gains in clinical tolerance [127].

Prospective interventional data further support this dissociation. In a phase II open‐label study involving 24 children (mean age 11.7 ± 3.3 years) with peanut allergy, dupilumab monotherapy resulted in substantial reductions in total IgE (−54%) and peanut‐specific IgE (−49%) after 24 weeks. Nevertheless, only 8.3% of participants met the primary endpoint of tolerating 444 mg of peanut protein, and no meaningful increase in the overall tolerated dose was observed. Skin prick test responses decreased by less than 10%, and basophil activation showed only modest changes. Notably, no exposure–response relationship was identified, suggesting that higher drug exposure did not correlate with improved clinical outcomes [128].

Dupilumab has also been evaluated as an adjunct to oral immunotherapy (OIT). In the randomized phase II trial (NCT03682770), the addition of dupilumab to peanut OIT modestly improved desensitization rates at the end of the up‐dosing phase compared with OIT alone (*p* < 0.05) in 148 children and adolescents aged 6–17 years. However, this benefit was not sustained during the maintenance phase and did not reach statistical significance at later time points (*p* = 0.21). Importantly, dupilumab did not significantly enhance the safety profile of OIT, as rates of anaphylaxis and other adverse events were comparable between groups [129].

Taken together, these findings indicate that while dupilumab exerts a clear effect on immunologic biomarkers in FA, particularly allergen‐specific IgE, this has not yet been translated into consistent short‐term clinical efficacy, either as monotherapy or in combination with OIT.

Across the conditions reviewed, the likelihood of clinical benefit appears to be influenced by the presence of T2 inflammatory biomarkers and related clinical features. Figure 3 summarizes a proposed framework for biomarker‐guided patient selection in emerging paediatric indications of dupilumab.

**FIGURE 3:**
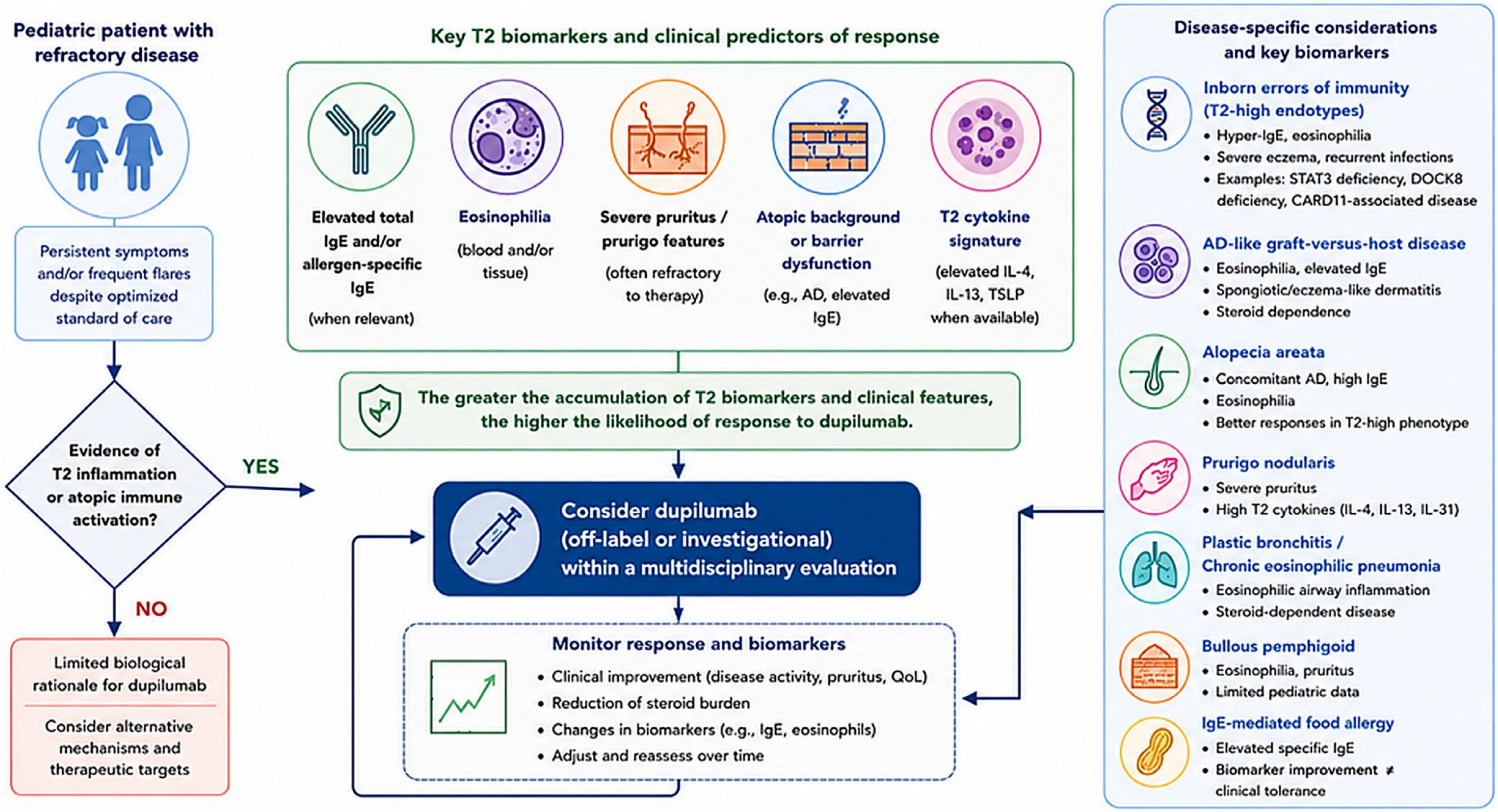
Biomarker‐guided patient selection for emerging paediatric indications of dupilumab. Proposed clinical approach to identify paediatric patients with evidence of T2 immune activation who may be candidates for investigational or off‐label dupilumab treatment (AD, atopic dermatitis; IgE, immunoglobulin E; IL, interleukin; IL‐4Rα, interleukin‐4 receptor alpha subunit; QoL, quality of life; T2, type 2; TSLP, thymic stromal lymphopoietin).

## 7 Conclusion

Dupilumab is increasingly being explored in paediatric conditions beyond approved indications, reflecting the expanding recognition of T2 inflammation across heterogeneous diseases. The current evidence, biological rationale, reported outcomes, and major limitations across emerging indications are summarized in Table 1.

**TABLE 1:** Emerging paediatric indications of dupilumab beyond current approvals: Biological rationale, clinical evidence, outcomes, and limitations.

Clinical condition	Biological rationale for IL‐4Rα blockade	Current level of evidence	Main reported clinical outcomes	Major limitations and unmet needs
AA	Subgroup characterized by T2 activation, elevated IgE, eosinophilia, and concomitant AD; increased IL‐4/IL‐13 signalling may contribute to disease expression	Small retrospective cohorts, case series, case reports; ongoing paediatric clinical trial (NCT05866562)	Clinically meaningful hair regrowth in approximately 70%–80% of reported paediatric patients with concomitant AD; greater responses associated with elevated IgE and lower baseline SALT scores	Most patients treated primarily for AD; efficacy in non‐atopic AA remains uncertain. Reports of paradoxical onset or worsening of AA suggest endotype‐specific responses
PN	Neuroimmune disease involving T2 cytokines (IL‐4, IL‐13, IL‐31), neuronal sensitization, substance P signalling, and barrier dysfunction	Paediatric evidence limited to case reports	Rapid reduction in pruritus, improvement in nodular lesions, sleep quality, and QoL; responses often observed within weeks	No controlled paediatric studies. Current paediatric evidence largely extrapolated from adult experience
BP	T2 cytokines contribute to eosinophilic inflammation, pruritus, and disease amplification in BP	Single paediatric case report	Complete remission of blistering and pruritus with corticosteroid‐sparing effect	Evidence restricted to isolated observations; no paediatric cohorts or prospective studies
IEI	Many IEIs exhibit marked T2 skewing, hyper‐IgE, eosinophilia, severe eczema, and barrier dysfunction	Numerous case reports and case series; nationwide multicenter cohort	Consistent improvement in eczema severity, pruritus, sleep, QoL, IgE levels, and corticosteroid requirements; favourable safety profile. Highest response rates reported in STAT3 deficiency and other T2‐dominant phenotypes	Marked genetic heterogeneity; lack of standardized outcome measures; limited long‐term follow‐up and biomarker validation
AD‐like GvHD	Selected patients display eosinophilia, elevated IgE, spongiotic dermatitis, and T2 immune signatures resembling AD	Several paediatric case reports and small case series	Rapid improvement in eczema and pruritus; facilitated tapering or discontinuation of systemic immunosuppressive therapies; durable responses reported	Absence of standardized diagnostic criteria; no prospective trials; unclear applicability to non‐T2 GvHD phenotypes
PB	Eosinophilic airway inflammation and IL‐4/IL‐13‐driven immune activation identified in inflammatory bronchial casts	Single paediatric case report	Sustained remission of eosinophilic PB with prevention of recurrent cast formation and improved lung function	Anecdotal evidence only; no data regarding patient selection, biomarkers, or long‐term outcomes
CEP	Steroid‐dependent eosinophilic pulmonary inflammation represents a biologically plausible target for IL‐4Rα blockade	Single paediatric case report	Clinical, radiological, and functional remission with successful withdrawal of concomitant immunosuppressive therapy	Evidence limited to one case; efficacy and optimal positioning within treatment algorithms remain unknown
IgE‐mediated FA	IL‐4/IL‐13 blockade reduces allergen‐specific IgE production and may theoretically facilitate immune tolerance	Retrospective cohorts and phase II clinical trials (monotherapy and adjunct to OIT)	Significant reductions in total and food‐specific IgE; modest and inconsistent effects on desensitization outcomes	Improvements in biomarkers have not translated into consistent clinical tolerance. Role in routine FA management remains undefined

Abbreviations: AA, alopecia areata; AD, atopic dermatitis; BP, bullous pemphigoid; CEP, chronic eosinophilic pneumonia; FA, food allergy; GvHD, graft‐versus‐host disease; IEI, inborn errors of immunity; IgE, immunoglobulin E; IL, interleukin; IL‐4Rα, interleukin 4 receptor alpha subunit; OIT, oral immunotherapy; PB, plastic bronchitis; PN, prurigo nodularis; QoL, quality of life; SALT, Severity of Alopecia Tool; T2, type 2.

Based on the available evidence, the conditions most likely to benefit from dupilumab are those with a clear T2‐dominant phenotype, including IEI characterized by severe atopy, hyper‐IgE, eosinophilia, and refractory AD‐like inflammation, as well as selected cases of AD‐like GvHD with eosinophilia, elevated IgE, severe pruritus, and steroid‐dependent or steroid‐refractory disease. In these settings, dupilumab may provide clinically meaningful disease control and a potential steroid‐sparing effect.

A second group includes diseases in which dupilumab shows promise but requires confirmation in larger paediatric cohorts, such as AA associated with concomitant AD or other T2 biomarkers, PN with severe atopic pruritus, and selected eosinophilic respiratory disorders such as PB and CEP. In these conditions, reported responses are encouraging, but the available evidence remains limited to small series or isolated cases.

Finally, for conditions such as paediatric BP and IgE‐mediated FA, the biological rationale is plausible but current clinical evidence remains insufficient or inconsistent. In FA, reductions in total and allergen‐specific IgE have not yet been translated into consistent clinical tolerance, either as monotherapy or in combination with OIT.

Overall, dupilumab appears most clinically relevant in paediatric diseases with demonstrable T2 immune activation, particularly when conventional therapies are ineffective or associated with substantial toxicity. However, current evidence remains limited by small sample sizes, heterogeneous populations, variable outcome measures, and short follow‐up. Although dupilumab has generally shown a favourable safety profile across the conditions reviewed, available safety data remain limited by the predominance of case reports and small observational cohorts, precluding definitive conclusions regarding rare or long‐term adverse events. Prospective studies are needed to define patient selection, predictive biomarkers, optimal timing of treatment, long‐term safety, and disease‐specific efficacy.

## Author Contributions

Conceptualization, G.L.M.; methodology, S.F.R., and A.L.; software, S.M.; validation, A.L., and G.L.M.; formal analysis, M.D.A.; investigation, A.C., S.P., and M.T.; resources, D.D., and E.M.L.; data curation, A.M., and V.B.; writing‐original draft preparation, S.F.R., and A.L.; writing‐review and editing, G.L.M.; visualization, S.F.R., A.C., D.D., A.M., E.M.L., S.P., M.T., M.D.A., S.M., V.B., A.L., and G.L.M.; supervision, A.L. and G.L.M. All authors have read and agreed to the published version of the manuscript.

## Funding

The authors have nothing to report.

## Conflicts of Interest

The authors declare no conflicts of interest.

## Data Availability

Data sharing not applicable to this article as no datasets were generated or analysed during the current study.
